# Cultivating marine bacteria under laboratory conditions: Overcoming the “unculturable” dogma

**DOI:** 10.3389/fbioe.2022.964589

**Published:** 2022-08-17

**Authors:** Carlos J. C. Rodrigues, Carla C. C. R. de Carvalho

**Affiliations:** ^1^ Department of Bioengineering, iBB-Institute for Bioengineering and Biosciences, Instituto Superior Técnico, Universidade de Lisboa, Lisbon, Portugal; ^2^ Associate Laboratory I4HB—Institute for Health and Bioeconomy, Instituto Superior Técnico, Universidade de Lisboa, Lisbon, Portugal

**Keywords:** metataxonomy, metagenomics, culturomics, biocatalyst, marine biotechnology, isolates

## Abstract

Underexplored seawater environments may contain biological resources with potential for new biotechnological applications. Metagenomic techniques revolutionized the study of bacterial communities but culture dependent methods will still be important to help the biodiscovery of new products and enzymes from marine bacteria. In this context, we promoted the growth of bacteria from a marine rock pond by culture dependent techniques and compared the results with culture independent methods. The total number of bacteria and diversity were studied in different agar plate media during 6 weeks. Agar plate counting was of the same order of magnitude of direct microscopy counts. The highest efficiency of cultivation was 45% attained in marine agar medium. Molecular analysis revealed 10 different phyla of which only four were isolated by the culture dependent method. On the other hand, four taxonomic orders were detected by cultivation but not by the molecular technique. These include bacteria from the phyla Bacillota and Actinomycetota. Our study shows that it is possible to grow more than the traditionally considered 1% of bacteria from a seawater sample using standard agar plate techniques and laboratorial conditions. The results also demonstrate the importance of culture methods to grow bacteria not detected by molecular approaches for future biotechnological applications.

## Introduction

The knowledge about the role of microorganisms in the different habitats on earth improved tremendously since the first observations showing their existence in the XVII century by Robert Hooke and Antonie van Leeuwenhoek (Gest, 2004). The following two centuries were characterized by the use of mainly the microscope for the classification of microorganisms (Summers, 2009). Major technological and scientific advances were achieved during the “golden age” of bacteriology, including the development of the solid media culture technique by Robert Koch, Fannie Hesse, Julius R. Petri and other scientists (Blevins and Bronze, 2010; Bonnet et al., 2019). This latter advance, which is routinely used today in microbiology laboratories, created the division between the cultured and “uncultured” world of microorganisms. The growth of isolates in pure culture contributed to the “unculturability” of many species since it prevents the cells from sharing metabolites and signaling compounds that they would share with neighbors in a natural community (Davis, 1921; Kearney et al., 2021).

In 1932, Razumov compared the number of bacteria from aquatic habitats obtained by a direct method calculation using a microscope and by Koch’s plate count technique (Razumov, 1932). More cells were counted, by several orders of magnitude, with the microscope method than by the plate count method in oligotrophic and mesotrophic aquatic habitats. Similar results were reported in samples of sea water by Jannasch and Jones in 1959 (Jannasch and Jones, 1959). They compared two direct microscope methods with five different culture methods. Some explanations presented for the anomaly included the presence of bacteria in aggregates, presence of non-viable cells and the selective media used. Some decades later, in 1982, Staley et al. reported that only 0.1–1% of the total bacteria determined by microscopic counts in lake water samples could be enumerated by culture plate techniques (Staley et al., 1982). Staley and Konopka thus made a significant contribution to the famous “great plate count anomaly” in 1985 by stating that “as a general rule we have found that the maximum recovery of heterotrophic bacteria is 1% of the total direct count using plating procedures or other viable enumeration methods from a variety of oligotrophic to mesotrophic aquatic habitats” (Staley and Konopka, 1985). However, the low number of bacteria probably resulted from the failure to mimic the conditions (such as nutrient type and concentration, pH, osmotic conditions, and temperature), and knowledge on targeted species of the habitat (Staley and Konopka, 1985; Alain and Querellou, 2009; Vartoukian et al., 2010; Stewart, 2012).

The implementation of molecular techniques allowed the identification of uncultured bacteria. In 1990, Giovannoni et al. studied the diversity of the Sargasso Sea bacterioplankton using PCR amplification of 16S rRNA genes, sequencing and identification of the source of these genes by phylogenetic analysis (Giovannoni et al., 1990). Ward et al. used the same approach to study the microbial community in a hot spring (Ward et al., 1990). Molecular techniques thus allowed to access the hidden microbial diversity of environmental habitats (Amann et al., 1995).

In the beginning of the XXI century, metagenomics allowed the understanding that the unknown microbial diversity is immense, and the classification of yet-to-be cultured microbes (Venter et al., 2004; Schloss and Handelsman, 2005; Chauhan, 2019). Whole-genome shotgun sequencing allows the generation of millions or billions of base pairs of non-redundant sequences, their annotation and analysis to determine their gene content, diversity and relative abundance of the organisms from environmental samples (Venter et al., 2004; Schloss and Handelsman, 2005).

Since “unculturable microbes” does not mean that they are “not culturable”, but that (in most cases) no one has attempted to grow them, the terminology “not-yet-cultured” or “yet-to-be cultured” seems more appropriated (Gest, 2008). Many of the phylogenetically new taxa discovered by culture independent approaches lack cultured representatives (Lloyd et al., 2018). However, their cultivation in laboratory is extremely important to study their physiology and their role in the environmental community (Lloyd et al., 2018; Lewis et al., 2021). For example, the study of a cultured extremophile bacterium resulted in the discovery of a previously unknown reversible tricarboxylic acid (TCA) cycle only when genomic, enzymatic and metabolomics analyses were used (Nunoura et al., 2018). Additionally, culture techniques proved able to identify species that were not detectable by metagenomic and metaxonomy approaches (Donachie et al., 2007) and could be superior to these approaches in the case of microbiota studies (Dubourg et al., 2013; Pfleiderer et al., 2013). This evidence aroused the revival and importance of culture techniques for future projects (Lagier et al., 2015; Overmann et al., 2017; Thrash, 2019). In fact, culturomics, which consists on the application of a large number of culture media and conditions, has been shown to be very important e.g. in studies of human microbiota during the last few years (Lagier et al., 2015; Lagier et al., 2016).

New and innovative culture techniques have been developed and used to grow novel microorganisms *in vitro*, including co-culture with other bacteria, density-based separation, dilution-to-extinction, and the use of selective inhibitors and of diffusion chambers (Kaeberlein et al., 2002; Nichols et al., 2010; Stewart, 2012; Kearney et al., 2021; Lewis et al., 2021). Nevertheless, some of these techniques still need further implementation across different laboratories and increased reproducibility. The implementation of simple alterations to the traditional culture methods could also increase the number of cultured bacteria. Recently, Jung et al. compared diffusion chambers, dilution-to-extinction culture and modified agar preparation to grow bacteria from an intertidal zone (Jung et al., 2021). The simple separation of media ingredients during the sterilization process increased the number of different bacteria grown. The effort implemented in the advanced cultivation methods permitted to culture a total of 201 novel species that did not appear following standard direct plating. Moreover, the implementation of these techniques allowed the identification of 45% of the total diversity found by culture-independent techniques. In another study, Demko et al. analysed the microbial diversity on marine tropical sediments, and the authors were able to culture 3% of the >800 genera detected by culture independent methods using only two culture media and three dilutions of the samples (Demko et al., 2021). Furthermore, 39 genera could only be detected by cultivation.

It is worth mentioning that the traditional methods of plate counting were not originally designed to isolate and grow all species from an environmental sample, but to easily handle and study a determined microorganism in pure culture under laboratory conditions. Furthermore, “the great plate count anomaly” was originated from the difference between the number of cells observed under the microscope and the number of cells determined by plate count techniques, in mesotrophic and oligotrophic habitats, as previously mentioned. The dogma was eventually generalized and it is now accepted that less than 1% of bacteria grows under laboratory conditions.

In the present study, a rock pond was used as sampling site because we have been able to find efficient biocatalysts in this type of marine environment (Rodrigues et al., 2017; Rodrigues and de Carvalho, 2022), which is characterized by high salt concentration and UV-exposure. The aim of the study was 1) to compare the number of cells that could be grown under laboratorial conditions with the number of cells observed by fluorescence microscopy, and 2) to compare the isolates obtained by cultivation methods with those identified by metataxonomy. The ultimate goal was to obtain a culture collection of bacteria that could be easily screened for enzyme activities and/or the production of commercially interesting secondary metabolites which are difficult to assess by genetic sequences.

## Materials and methods

### Sample collection and processing

Water sample from a rock pond was collect on 13 October 2020. The rock pond (coordinates: 38.715,524, −9.483,665; 38°42′55.9″N 9°29′01.2″W) is located near Cabo Raso and Guincho beach, on the west coast of Portugal. The pond is on a rocky shoreline with low cliffs, with several deep and narrow incisions, exposed to Atlantic swell and waves. The water in the rock pond was at 20°C, the air was also at 20°C and the seawater was at 16°C. The concentration of salts in the water sample determined by the evaporation method was ca. 60 g/L. The water collected was immediately transported to the laboratory and filtered through a paper filter grade 2 (Whatman, United Kingdom) to remove copepods and microalgae aggregates visible under the naked eye in the water sample. The filtered water was used in the same day for the culture dependent techniques and microscopy analysis, whilst filters for culture independent approaches were preserved at −16°C.

### Agar plate culture techniques and identification

The growth of microorganisms from the environmental sample was promoted in multiple agar media under laboratory conditions. The media used were the following: tryptic soy broth (TSA), potato dextrose agar (PDA), Middlebrook 7H9 broth (M7H9) all from Becton, Dickinson and Company, United States; blood agar (BA), thioglycollate broth with resazurine (THIO), Mueller Hinton broth (MH), sea salts (SS), cyanobacteria BG-11 freshwater solution (BG11; for marine species 10 g/L sodium chloride and 1 μg/L vitamin B12 were added), Guillard’s (F/2) marine water enrichment solution (GMW), all from Sigma-Aldrich, United States; and marine agar (MA) from Condalab, Spain. The media TSA, PDA, BA, THIO and MH were also used diluted 10 and 100-fold, as we previously suggested (Rodrigues et al., 2017). The media without agar in their composition or media diluted were supplemented with agar up to 15 g/L. Sea Salts (SS) medium was used at a concentration of 38 g/L, and it was supplemented with 5 g/L of glucose for the sea salts with glucose (SSG). The composition of the mineral medium agar (MMA) is listed in (Cortes and de Carvalho, 2015) and it was supplemented in the present study with 5 g/L glucose and 3.5 g/L yeast extract. The media were sterilized by autoclave (120°C for 20 min) and poured into square dishes (120 mm × 120 mm × 17 mm, from Greiner GmbH, Pleidelsheim, Germany) under sterile conditions. The plates were inoculated by spreading 50 µl of water sample and incubated at room temperature. Colony forming units (CFUs) in the agar plates were monitored for 6 weeks. Colonies with distinct phenotype were picked and streaked into a new agar plate to promote their growth as pure culture. The pure bacterial cultures were grown in TSA and identified using the Sherlock^®^ Microbial ID System from MIDI, Inc. as previously described (Rodrigues et al., 2017). Identification of isolates 10, 13, 18, 20, 31, 42, 49, and 55 were also performed by 16S rRNA sequencing. The extraction of DNA from the isolates was executed with DNeasy Powerwater kit from Qiagen (Hilden, Germany), according to the manufacturer’s instructions. Sanger sequencing of the 16S rRNA fragments from the PCR amplification of the variable regions V1 to V9 was done by Stab Vida (Lisbon, Portugal). Alignment of sequences was done to regenerate the consensus sequence which was compared with those available at the GenBank database of the National Center for Biotechnology Information (NCBI, http://www.ncbi.nih.gov), by using the advanced BLAST search tool.

### Microscopy techniques

The calculation of the number of cells in the collected sample was performed using a Neubauer chamber (Hirschmann, Germany) by both visual counting and image analysis. A cell culture contamination detection kit (Invitrogen™, ThermoFisher Scientific, United States), which stains yeast and other fungi, Gram-positive and Gram-negative bacteria by slide preparation, was used to determine the number of bacterial cells in the sample. Cell viability was determined by staining the cells with the LIVE/DEAD™ *Bac*Light™ Bacterial Viability Kit (Invitrogen™, ThermoFisher Scientific, United States). Both kits were used according to the manufacturer’s instructions.

To further determine the number of cells, 100 ml of water were filtered through a membrane black filter with a white grid (ME 25/31 ST, 0.45 µm, from Whatman™) and then through a white filter with a black grid (ME 24/21 ST, 0.2 µm, from Whatman™). Cells were stained with SYTO™ nine green fluorescent nucleic acid stain (Invitrogen ™, ThermoFisher Scientific) to determine their number.

Both direct observations with a microscope and image analysis were performed to quantify the number of bacterial cells in the water sample, using the Neubauer chamber and the filters. Observations were made using an Olympus CX40 microscope equipped with an Olympus U-RFL-T burner and the following mirror cube unit filters: U-MWU (excitation filter: BP330–385; barrier filter: BA420); U-MWB (excitation filter: BP450–480; barrier filter: BA515); U-MWG (excitation filter: BP510–550; barrier filter: BA590). Images were collected by an Evolution™ MP 5.1 CCD color camera using the acquisition software Image-Pro Plus (both from Media Cybernetics, United States). Cell number and viability were calculated by image analysis using Visilog 5 (Noesis SA, France) as previously described (de Carvalho et al., 2003).

### Culture independent approach (metataxonomy)

The 100 ml water sample was filtered (Whatman ME 24/21 ST 0.2 µm) and the DNA extracted with DNeasy Power Water kit (Qiagen). Microbial communities were characterised by library construction using Illumina 16S Metagenomic Sequencing Library protocol, and the DNA libraries were sequenced with MiSeq Reagent Kit v3 in the lllumina MiSeq platform, using 300 bp, by Stab Vida (Lisbon, Portugal). The PCR libraries were prepared by amplification of the V3 and V4 regions of the 16S rRNA gene. The raw sequence data obtained by Stab Vida was processed using QIIME2 v2020.8 (Caporaso et al., 2010) and denoised by the DADA2 plugin (Callahan et al., 2016). A taxonomical classification of the 170 unique features (OTUs) was performed using the SILVA database (release 138 QIIME) (Quast et al., 2013), trained using scikit-learn, with a clustering threshold of 97% similarity. Only OTUs containing at least 10 sequence reads were considered significant for classification purposes.

### Analysis

The software R (version 4.1.2, from The R Foundation for Statistical Computing) was used for data analysis. The data from the PCA analysis was treated using the method Euclidean to generate the circular dendrogram. The number of optimal clusters used in the Euclidean analysis was determined using the Elbow method. The package nVennR (Pérez-Silva et al., 2018) was used to create the proportional Venn diagram using the taxonomic orders of isolates.

## Results

### Total number of cells in the bacterial community

Both direct observations with a microscope and image analysis were performed to quantify the number of bacterial cells in the water sample. Using a Neubauer chamber it was possible to determine that the number of bacterial cells in the sample was 4.5 × 10^4^ cells/mL (data not shown). The viability of cells in the sample was determined using a viability kit with fluorescent dyes: 93.6% of cells were viable, which corresponds to 4.2 × 10^4^ viable cells/mL. This number was confirmed by observing and counting cells on the surface of filters with grids which had been used to filter 100 ml of water sample. Additionally, the cell number estimated by direct counting using the Neubauer chamber were in agreement with the number of cells determined by supervised image analysis of cells stained with the viability kit.

The number of viable cells in the water sample obtained by microscopy was compared with the number of cells determined by the agar plate counting method. For that, we prepared agar plates with 17 media compositions (Table 1). Colony forming units (CFUs) in each agar plate were monitored for 6 weeks and counted. The presumption that one colony is the result of a single cell growth was used. However, observation of the samples by fluorescence microscopy showed also the presence of clusters of bacterial cells which should result in a single colony.

**TABLE 1:** Media composition and dilution factor used to promote bacterial growth in agar plates and respective number of colony forming units (CFU), number of distinct phenotypes observed on the colonies formed, and percentage of bacterial isolates identified by their fatty acid (FA) profile. The error associated to CFU counting was less than 5%. * in plates prepared with commercial media broth, 15 g/L of agar was added to prepare the solid media.

Media code	Media*	Dilution factor	CFU	Colony phenotypic diversity	Identified by FA profile (%)
BA1	Blood agar	1	0	0	0
BA10		10	12	5	20
BA100		100	20	3	50
BG11	Cyanobacteria BG-11 freshwater solution	1	3	1	0
GMW	Guillard’s (F/2) marine water enrichment solution	1	11	4	25
MA	Marine agar	1	940	14	13
MH1	Mueller hinton broth	1	0	0	0
MH10		10	31	6	43
MH100		100	14	3	33
M7H9	Middlebrook 7H9 broth	1	10	6	43
MMA	Mineral medium	1	0	0	0
PDA1	Potato dextrose agar	1	0	0	0
PDA10		10	3	1	0
PDA100		100	20	5	17
SS	Sea salts	1	409	4	0
SSG	Sea salts with glucose	1	12	5	0
TSA1	Tryptic soy agar	1	0	0	0
TSA10		10	22	8	30
TSA100		100	35	10	27
Thio1	Thioglycollate broth w/Resazurine	1	1	1	100
Thio10		10	24	10	33
Thio100		100	28	5	29

The marine agar (MA) and sea salts (SS) media allowed the highest number of CFUs compared with the remaining solid media used (Table 1). In the MA, 1.88 × 10^4^ CFU/mL were attained, which is in the same order of magnitude of the microscope observations. This corresponds to a cultivation efficiency of 44.6%, i.e., 44.6% of the cells observed under the microscope resulted in cell colonies in MA. The number of CFUs observed in the SS agar media was 8.18 × 10^3^ cells/mL, corresponding to 19.4% of cultivation efficiency. Each remaining media allowed cultivation efficiencies lower than 2%.

### Culture-dependent methods to assess the bacterial community

The growth of the bacterial cells present in the rock pond was promoted in laboratory using the conventional spread-plate method but using a culturomics approach. The same sample was seeded on a large number of agar plates with different media compositions, as previously mentioned (Table 1). In addition to the CFUs, the phenotypic description of each colony in each agar plate was monitored for 6 weeks. The highest number of both CFUs and phenotypes were attained following cultivation in MA medium, reaching the number of 940 CFUs and 14 phenotypes, respectively (Table 1). SS medium also promoted a high number of CFUs (409), but a lower phenotypic diversity. With the exception of the Mueller-Hinton (MH) medium, the number of CFU increased with the media dilution factor: medium diluted 100-fold promoted the growth of more cells than medium diluted 10-fold, and with undiluted Blood agar (BA), MH, Potato dextrose agar (PDA) and Tryptic soy agar (TSA) no colonies were observed.

The isolates of each phenotype were initially identified by their lipid profile by the Sherlock™ Microbial ID System (MIS). The automated system compares the fatty acids of the membrane of the isolates grown in the laboratory with those in the MIS library (Kunitsky et al., 2006). In this study, the system could identify in general ca. 28% of the isolates (Table 1). The relatively low percentage of identified isolates could be ascribed to the low number of membrane profiles from marine bacteria found in the library, although the system has been used to identify seafood and halophilic bacteria (Surve et al., 2012; Morey et al., 2013).

**FIGURE 1:**
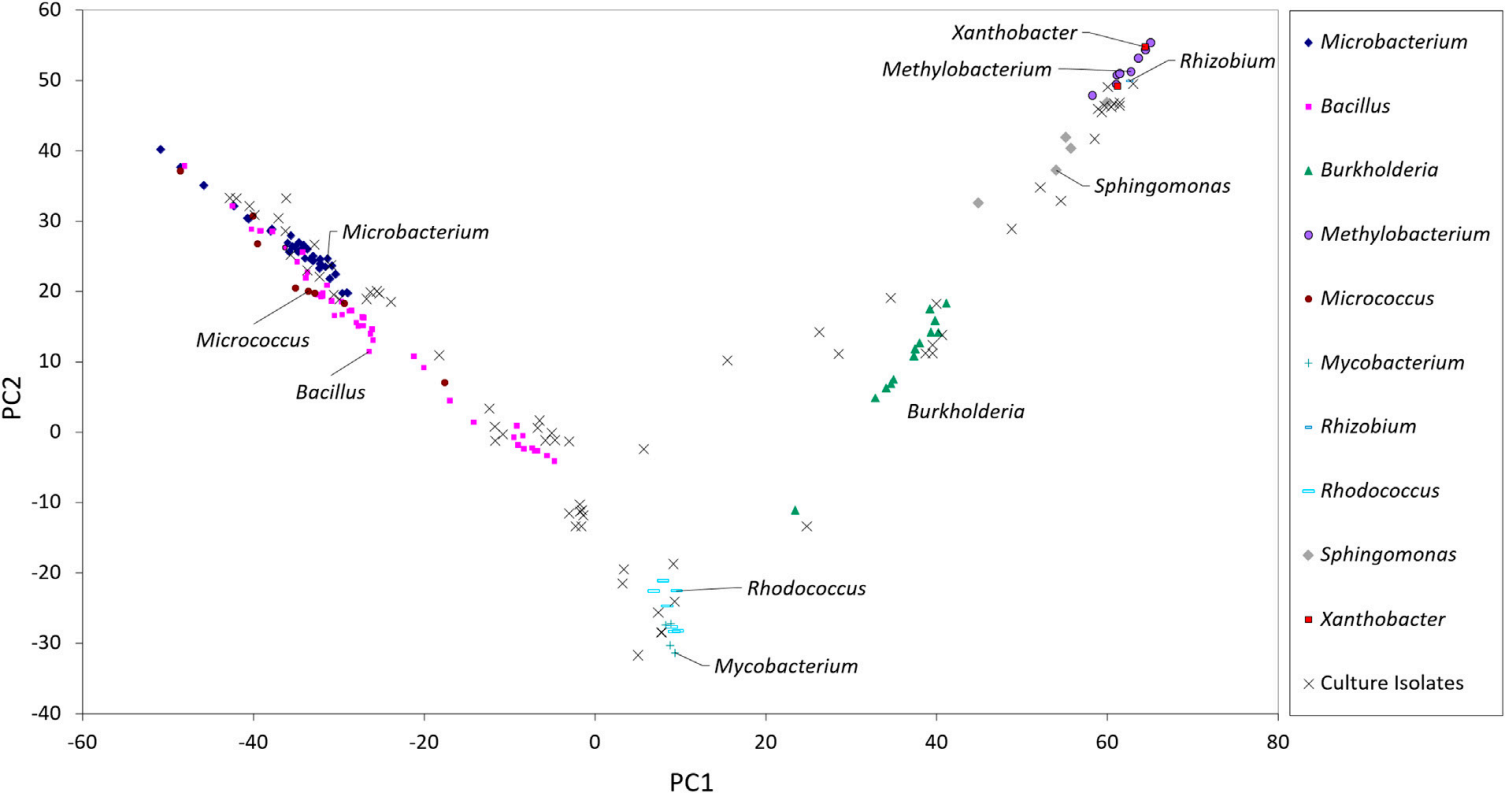
Scores of the sampled isolates along the first two axes resulting from PCA of fatty acid composition of selected bacterial genera present in the Sherlock™ MIS library.

**FIGURE 2:**
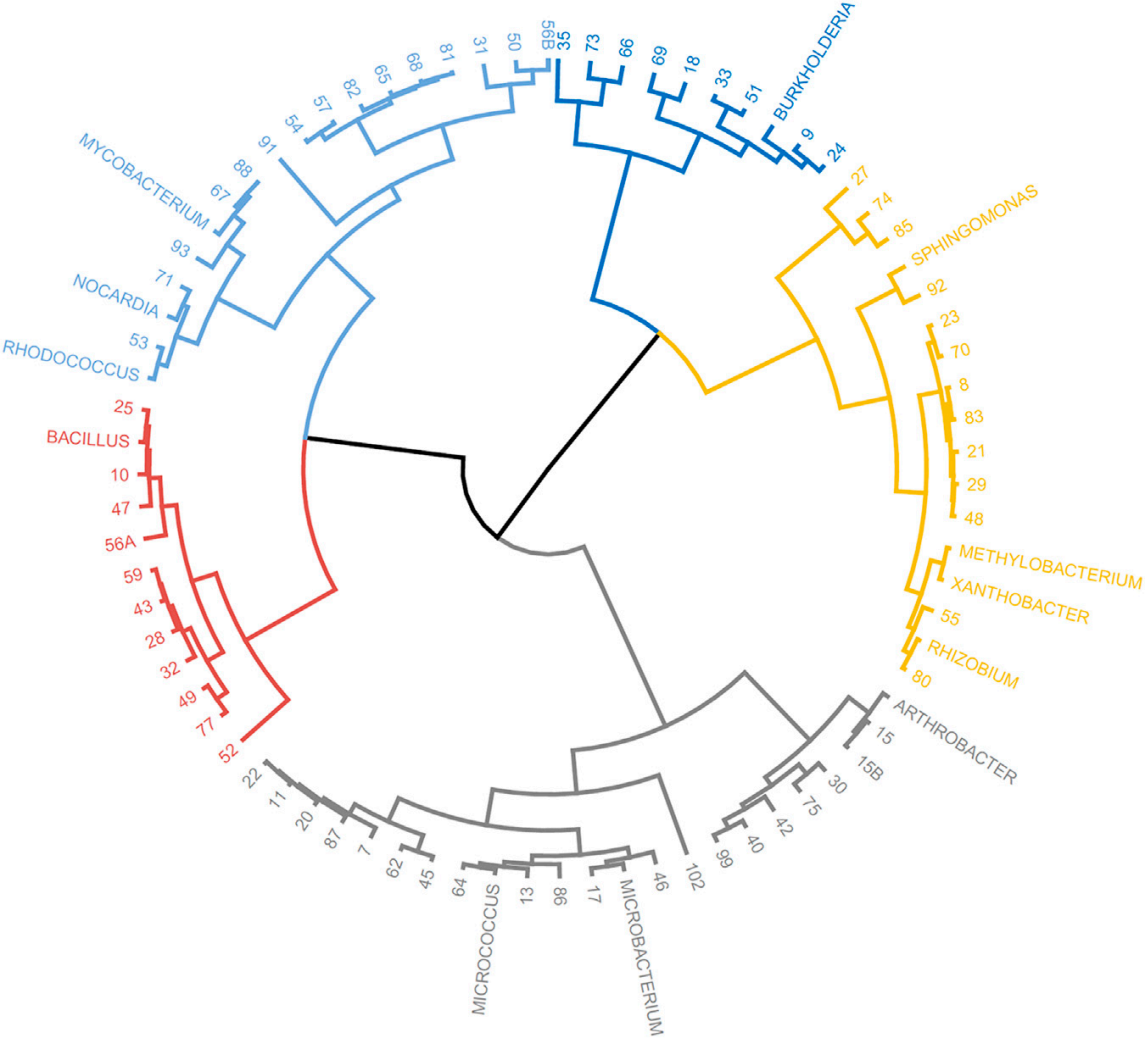
Circular dendrogram of the phylogenetic tree obtained by Euclidian analysis of the fatty acid profiles of selected bacterial genera present in the MIS library and that of the unidentified isolates from the rock pool grown in the laboratory.

The taxonomic genera of the isolates may be further investigated using their fatty acid profiles and the proximity to known genera (de Carvalho and Caramujo, 2014). Principal component analysis (PCA) using the fatty acid profiles of the isolates cultured in the laboratory and the profiles of specific genera deposited in the database was performed (Figure 1). The analysis showed the distribution of the isolates along different genera and that PC1 could separate the isolates: Gram-positive isolates were located at PC1 values lower than 10 whilst Gram-negative isolates were located at PC1 values higher than 20. Using the same data, a Euclidean analysis was used to create a circular phylogenetic tree that resulted in 4 clusters (Figure 2). The number of “natural” clusters or *k* was determined by the Elbow method (Thorndike, 1953), as suggested by Czech and Stamatakis (Czech and Stamatakis, 2019).

To assess if the positioning of the isolates not identified by the Sherlock™ MIS in Figures 1, 2 was correct, several isolates were selected and their 16S rRNA was sequenced. The isolates analysed were found to belong to the genera *Brachybacterium, Janibacter, Leifsonia, Mesobacillus, Mucilaginibacter, Paraburkholderia, Pseudarthrobacter, Rhizobium, and Williamsia* (Table 2 and neighbor joining tree as Supplementary Figure S1). When comparing the location of the isolates according to the fatty acid profile in the dendrogram with the closest species according to the 16S rDNA sequencing (Table 2 and Supplementary Figure S1 in comparison with Figure 2), the results indicate that the fatty acid profile can be used to successfully infer about the phylum, and in some cases the genus, to which the isolate belong. For example, in the case of isolate 55, it appears next to a *Rhizobium* strain present in the database (Figure 2) and it presented the highest homology by 16S rRNA sequencing with a *Rhizobium multihospitium* in GenBank (Table 2). The same may be observed for isolate 10 which was identified as belonging to *Mesobacillus* and it is located close to *Bacillus* in the dendrogram. In the case of isolate 18, a certain proximity to the genus *Burkholderia* was suggested by the fatty acid profile and by the 16S rRNA sequencing since the isolate presented 100% similarity with a *Paraburkholderia fungorum* in GenBank*.* Both the genus *Burkholderia* and *Paraburkholderia* belong to the phylum Pseudomonadota (formely Proteobacteria).

**TABLE 2:** Suggested species of selected isolates by consensus sequence BLASTed againt NCBI nucleotide database.

Sample ID	Suggested species	Proximity (%)	Phylum
10	*Mesobacillus subterraneus*	99.85	Bacillota
13	*Pseudarthrobacter phenanthrenivorans*	100	Actinomycetota
18	*Paraburkholderia fungorum*	100	Pseudomonadota
20	*Leifsonia shinshuensis*	99.93	Actinomycetota
31	*Williamsia muralis*	99.62	Actinomycetota
42	*Brachybacterium faecium*	99.33	Actinomycetota
49	*Janibacter hoylei*	100	Actinomycetota
55	*Rhizobium multihospitium*	100	Pseudomonadota

**FIGURE 3:**
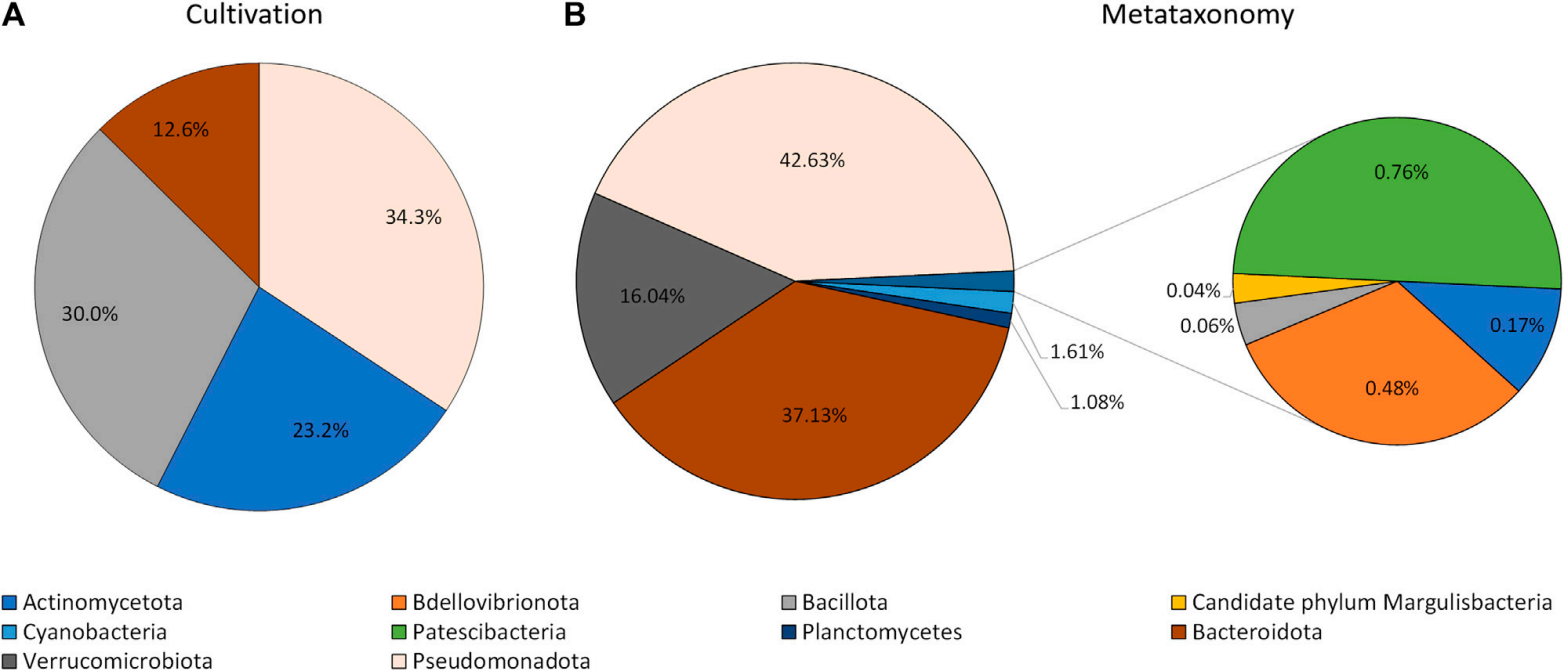
Phyla of bacteria identified using cultivation methods and the lipid profile **(A)**, and metataxonomy **(B)**.

The information collected by using the lipid profile and 16S sRNA analyses thus allowed the taxonomic characterization of isolates grown in the laboratory, by using the proximity of their fatty acid profile to known genus in the Sherlock™ MIS database, when the isolates were not automatically identified. The results indicate that isolates grown by cultivation methods belong to four phyla: Actinomycetota (formely Actinobacteria), Bacillota (formelly Firmicutes), Bacteroidota (formely Bacteroidetes), and Pseudomonadota (Figure 3). Thirty two isolates were difficult to sub-culture in TSA and could not be identified by their lipid profile. Of these, 37.5% were initially isolated in MA. The isolates which could not be grown in TSA represented 31% of all isolates but 86.8% of CFUs, with three isolates alone being responsible for 70.6% of CFUs grown in MA.

### Culture-independent methods to describe the bacterial community

The extraction of the total DNA from the water sample collected at the rock pond, followed by next generation sequencing (NGS) of the 16S amplicon, was used to determine the composition of the bacterial community present at the natural environment. The sample generated 120,148 raw sequence reads, which after denoising resulted in 170 Operational Taxonomic Units (OTUs). These were associated to a total of 10 different taxonomic phyla (Figure 3). The three major taxonomic groups identified in the bacterial community by metataxonomy were Pseudomonadota (42.63%), Bacteroidota (37.13%), and *Verrucomicrobiota* (16.04%). The latter phylum has been shown to be diverse by cultivation-independent studies but only a few members have been actually cultivated (Choo et al., 2007). In fact, 43.59% of the total OTUs in the present study correspond to uncultured bacteria and only 12.82% belong to known genera.

All four phyla identified using culture-dependent methods were identified using the molecular techniques, but no member of six of the phyla identified by metataxonomy could be identified “automatically” by their lipid profile because no representatives were present in the database. However, using the fatty acid profile of the isolates, it is possible to assess if cells from e.g. Verrucomicrobiota were present. By comparing the fatty acid profiles of the isolates of this study and type strains representing major lineages of the phyla previously published (Spring et al., 2016), it was possible to determine that isolate 93 and *Akkermansia muciniphila* are close, with 18:1 and 15:0 *anteiso* being the most abundant fatty acids (data not shown).

**FIGURE 4:**
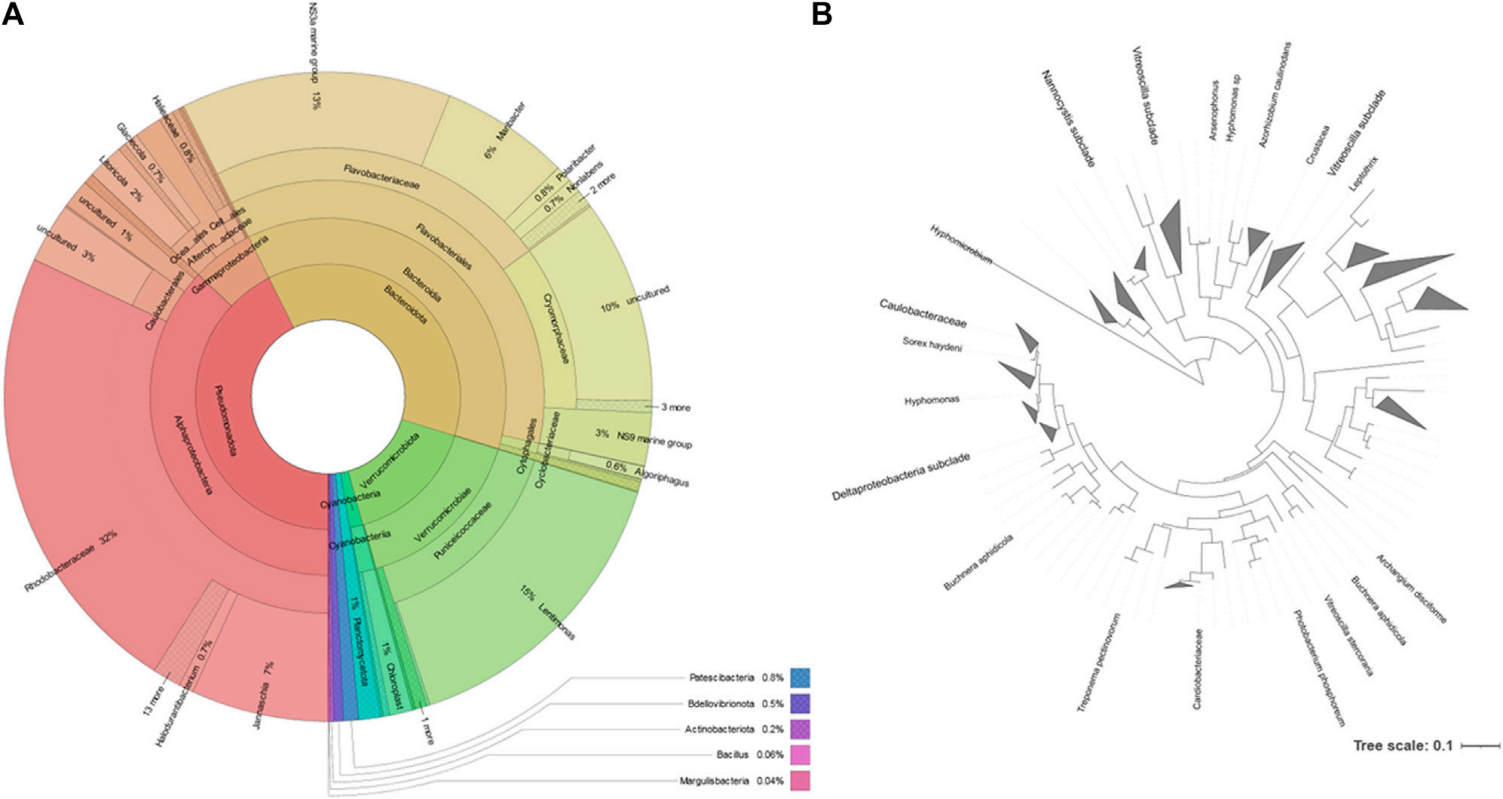
Taxonomic distribution of the microbial community of the rock pond according to the metataxonomic data. **(A)**: Krona chart, which should be read from the inside to the outside (Ondov et al., 2011). Colors indicate the taxonomic group to which the OUT was assigned and each ring of the chart represents a different taxonomic level. **(B)**: phylogenetic tree of the reads belonging to the Pseudomonadota phylum generated by the Interactive Tree of Life (iTOL) (Letunic and Bork, 2021); only bootstrap values > 0.6 are shown.

The most represented phylum, both by culture-dependent and independent methods, was the Pseudomonadota. This phylum represented 43% of the metataxonomy reads, with the family Rhodobacteraceae representing 32% (Figure 4). The second most represented phylum was Bacteroidota, which represented 37% of the reads. This phylum contained 13% of NS3a marine group bacteria and 6% Maribacter, belonging to the family Flavobacteriaceae. The family Cryomorphaceae represented 29% of the Bacteroidota identified but contained 10% reads corresponding to uncultured bacteria. The phylum Verrucomicrobiota represented 16% of the metataxonomy reads, with 15% corresponding to the genus *Lentimonas*. A simplified phylogenetic tree of life, according to Hug et al. (Hug et al., 2016), was constructed using the data for the Pseudomonadota (Figure 4).

**FIGURE 5:**
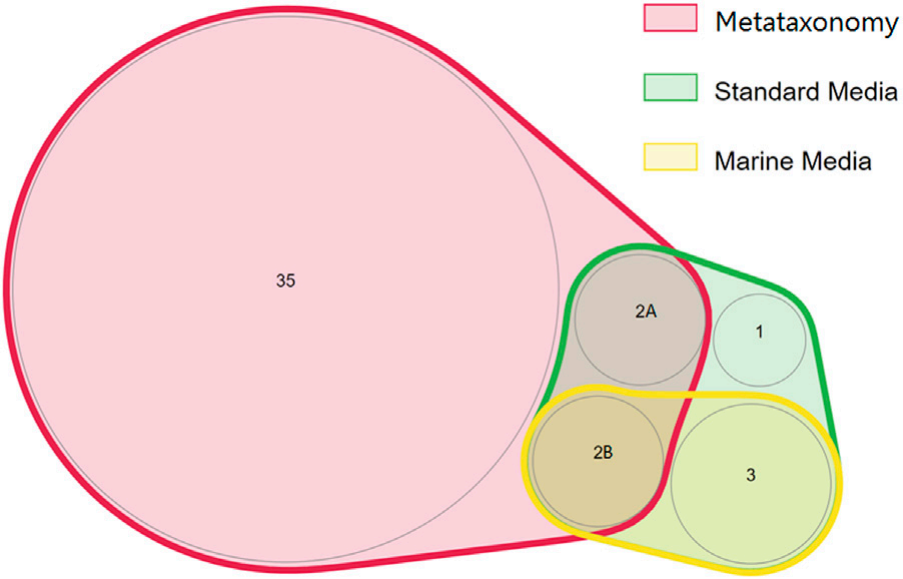
Proportionally scaled Venn diagram of microbial orders detected using culture-dependent and culture-independent approaches. “Marine media” correspond to BG11, GMW, MA, SS, and SSG media. “Standard media” corresponds to TSA, PDA, M7H9, BA, THIO, and MH media. The numbers inside each circle correspond to the number of orders detected. The orders are the following: 1—Sphingobacteriales; 2A- Caulobacterales, Enterobacterales; 2B—Micrococcales, Bacillales; 3—Hyphomicrobiales, Mycobacteriales, and Burkholderiales.

The phyla Bacillota and Actinomycetota were underrepresented in the bacterial community determined by metataxonomy, where they represented respectively 0.06 and 0.17% of the isolates. In comparison, with the culture-dependent approach, these phyla represented 20.6 and 27.8% of the isolates, respectively. At the order level of the bacterial community, the metataxonomy approach was able to identify 39 different taxonomic orders, of which 35 were only identified by this method, as shown in a proportionally scaled Venn diagram (Figure 5). Bacteria belonging to the orders Hyphomicrobiales, Burkholderiales, Mycobacteriales and Enterobacterales were only detected by the culture-dependent method. The orders Bacillales, Micrococcales, Sphingobacteriales and Caulobacterales were identified by metataxonomy and also isolated by the culture-dependent method used. The results referred to culture-dependent data were divided in cells grown using 1) marine culture media (BG11, GMW, MA, SS, and SSG) and 2) standard culture media (TSA, PDA, M7H9, BA, THIO, and MH). Marine culture media allowed the isolation of bacteria from orders Mycobacteriales, Bacillales, Burkholderiales, Hyphomicrobiales, and Micrococcales, but not members of the orders Sphingobacteriales, Caulobacterales and Enterobacterales. These were isolated in the standard culture media.

## Discussion

For a few decades, metagenomics was presented as the holy grail of microbiology as it allows access to “unculturable” microbial communities known as “dark matter” and thus the discovery of new molecules and identification of novel microbial members and activities (Handelsman et al., 1998; Schloss and Handelsman, 2005; Guazzaroni et al., 2010; Ferrer et al., 2018). In fact, metagenomics has provided enormous data sets and analysis tools, accessible to many research groups (Mitchell et al., 2017; Ferrer et al., 2018; Grossart et al., 2020). However, several problems in metagenomics have been identified, including: cloning and sampling bias, incorrect promoter sites; inaccurate estimation of species/taxon diversity because of 16S rRNA chimeras and artificial replicates; and reconstruction of artificial pathways (Keller and Zengler, 2004; Wooley and Ye, 2009; Sun et al., 2021). The immense knowledge provided by molecular techniques has not been successfully transferred to biotechnological products, mostly due to problems related to functional screening of putative genes (Mühling et al., 2013). The most effective approach to develop bioprocesses still remains the isolation and cultivation of microbial cells in laboratory (Joint et al., 2010; de Carvalho, 2017). However, growing microorganisms in laboratory is not an easy task and it is widely accepted that natural communities of bacteria have very low percentages of cultivation efficiency under laboratory conditions (Amann et al., 1995; Vartoukian et al., 2010). Curiously, it is often disregarded that the resolution of microbial communities by shotgun sequencing is also rather low, with dominant populations being responsible for a sequence assembly and community members present in low levels being missed (Warnecke and Hugenholtz, 2007). It is the aim of the present study to show that cultivation techniques may deliver more than the generally accepted dogma that defends that only between 0.1 and 1% of bacteria present in aquatic environments and soil are “culturable”. Culturomics has already challenged the dogma referring that the large majority of human microbiota is unculturable (Lagier et al., 2016; Diakite et al., 2020).

In the present study, we determined the number of cells in a marine rock pond by using both direct counts by microscopy and the spread-plate method on agar plates. The results showed a high cultivation efficiency using MA and SS, with *ca.* 45 and 19% of the number of cells resulting in CFUs, respectively. However, the majority of the other media tested resulted in cultivation efficiency lower than 2%. The results obtained contradict the generally accepted view that culture methods have poor growth efficiency: while the majority of media indeed only allowed the cultivation of ca. 2% of the cells counted by microscopy, MA and SS allowed a relatively high number of cells. The composition of MA was initially developed by Claude E. ZoBell in 1941 to allow the cultivation of the highest number of aerobic heterotrophic marine bacteria (Zobell, 1941). The medium contains nitrogen, vitamins, amino acids and minerals essential for growth of marine bacteria. The mixture of commercial sea salts used in SS was developed to mimic sea water composition. Compared to the other media tested, both MA and SS contain also higher concentration of sodium chloride (ca. 19 g/L whilst e.g. TSA contains 5 g/L and THIO 2.5 g/L). Since in the rock pond the cells were exposed to a high salt concentration, their cultivation was probably favored in media with higher salinity.

Curiously in most media tested, the number of CFUs observed increased with the dilution factor used, indicating that the marine bacteria preferred lower concentrations of nutrients and that a dilution of media helped the growth of certain strains. The main challenge in designing a cultivation media to mimic seawater rests in the accurate composition of dissolved organic carbon and trace elements (Zobell, 1941; Giovannoni and Stingl, 2007). It has been proposed that marine bacteria may represent a “low-nutrient-conditioned” phenotype which is usually perceived as an compulsory oligotrophic state (Schut et al., 1997). The coexistence of copiotrophic and oligotrophic bacteria in marine samples may result from fluctuations in nutrient availability in the marine environment and the presence of microniches: open waters are relatively nutrient depleted favouring oligotrophy but local variations may occur as result of e.g., upwelling of nutrient rich deep water or aggregation of particulate organic matter. Our data support the importance of selecting the appropriate media and time of incubation since sufficient time for the slow growers to appear in the plates must be provided. Oligotrophs are accustomed to low nutrient environment and need time to grow. This was the reason why we monitored the plates for 6 weeks: while in some media no new colonies were observed after the first week, in media such as the MA and M7H9 new colonies grew during the sixth week. Time and patience are mandatory to observe slow-growing marine bacteria. In fact, it has been suggested, by applying a model to oceanic metagenome data such as that available by the Global Ocean Survey data, that oligotrophs dominate the oceanic free-living microbial populations (Lauro et al., 2009). Oligotrophic bacteria were also found dominant in populations in the South China Sea and West Pacific Ocean by ^I4^C-MPN (Most Probable Number) method (Ishida et al., 1986).

Regardless of the relatively significant cultivation efficiency attained in the present study, there were still ca. 55% of the cells observed under the microscope that were not counted for in the agar plates. Three conditions may be responsible for this: 1) The methods used did not promote the growth of anaerobes, although Thio was one of the cultivation media tested: 2) The presence of viable but non-culturable (VBNC) bacteria in the water sample; 3) The assumption that one cell result in one colony in the agar plate but in fact aggregation of multiple cells could result in just one colony. The existence of VBNC cells, or dormant cells, in marine samples has been correlated with unculturability of indigenous bacteria (Oliver, 1993) and could be one of the most obvious characteristics of starvation-survival response (Schut et al., 1997). The factors that trigger the change from a dormant to a growing state are still poorly understood in bacteria from environmental habitats compared with infectious bacteria (Gonçalves and de Carvalho, 2016; Bodor et al., 2020).

Regarding the phenotypic diversity observed by the spread plating technique, the highest number of colony phenotypes was observed on MA where 14 different morphologies were observed (Table 1). However, only 13% of the diverse colonies on MA were identified by the fatty acid profile of their membranes. The number of cells identified was nonetheless higher in Thio10, DTSA100, MH10, M7H9 and DTSA10 than in MA, probably because the Sherlock™ library should contain mainly heterotrophs. Nevertheless, PCA indicated the proximity of the different isolates, even those not identified, with strains present in the library and 16S rRNA sequencing confirmed the identification of selected isolates.

An Euclidian analysis of the fatty acid profiles of the culture isolates and selected bacteria present in the Sherlock™ library indicated that the isolates grown in the laboratory belong to four different phyla (Figures 3, 4). They were Pseudomonadota (34.3%), Bacillota (30.3%), Actinomycetota (23.2%), and Bacteroidota (12.6%; Figure 3). On the other hand, ten different taxonomic phyla were identified in the bacterial community by metataxonomy: Pseudomonadota (42.6%), Bacteroidota (37.1%), Verrucomicrobiota (16.0%), and in lower percentages Actinomycetota (0.17%), Bdellovibrionota (0.5%), Bacillota (0.06%), Candidate phylum Margulisbacteria (0.04%), Cyanobacteria (1.6%), Patescibacteria (0.8%), and Planctomycetes (1.1%). Diversity of a bacterial community is better determined using culture independent techniques and it is one of the reasons for the success of these tools.

When comparing the phyla obtained by both methods, discrepancies in the percentages of their members in the community could be observed. The most notorious were observed with the phyla Actinomycetota and Bacillota which were highly represented by cultivation but nearly not detected by culture independent techniques. These two groups of Gram-positive bacteria have been found to be in greater proportion in bacterial marine communities than previously thought (Gontang et al., 2007; Leiva et al., 2015). Previous work carried out in our laboratory also showed significant abundance of Gram-positive isolates using culture dependent methods, with the genus *Bacillus* presenting the highest number of strains (13.2% if the isolates (Rodrigues et al., 2017). Several species of the phylum Bacillota produce endospores which protect them under harsh conditions and enable them to live in a myriad of environments. However, endospores are resilient to several traditional methods of DNA isolation, and only few endospore formers have been found in four metagenomic datasets although this bacterial phyum is the second most abundant (Filippidou et al., 2015). In fact, DNA extraction and PCR amplification has been shown to lead to larger bias than those due to sequencing and classification (Brooks et al., 2015).

In the present study, we isolated four taxonomic orders not detected by metataxonomy using standard culture dependent methods (Figure 4). As reported by the scientific community, culture independent and culture dependent methods do not detect the same community and the overlap in some habitats could be minimal (Donachie et al., 2007). The ideal situation is probably the conjugation of both dependent and independent culture methods to assess the “dark matter” of yet-to-be cultured marine bacteria (Öztürk et al., 2013; Solden et al., 2016).

Actinomycetota and Bacillota are renowned to produce a diverse range of bioactive metabolites. A bacterium belonging to the Bacillota phylum, *Bacillus amyloliquefaciens* MTCC 12716, was recently isolated by cultivation-dependent methods from marine macroalgae from an intertidal habitat and found to have antibacterial activities against multidrug-resistant bacteria (Kizhakkekalam and Chakraborty, 2020). The understanding of the complete physiology and metabolism of a bacterium, the production of secondary metabolites, and the study of how cells adapt to different conditions is only possible currently by cultivation. The isolation and cultivation of microorganisms is still a fundamental pillar of microbiology and biotechnology, and to find novel marine molecules. For example, Christensen and Martin reported several phyla of bacteria from ocean sediment samples and associated to the marine sponge *Halichondria panicea* with great potential for new drugs using culture dependent methods (Christensen and Martin, 2017). We also would like to emphasize the importance of subcultivation in laboratory. The MA medium had 31% of the isolates that escaped identification because they were difficult to subculture in TSA. As highlighted by Overmann *et al.*, it is extremely important to improve subcultivation, purification, and preservation techniques of fastidious bacteria to uncover microbial diversity (Overmann et al., 2017).

To conclude, our study shows that standard agar plate techniques can allow the growth of bacteria not detected by molecular techniques and to obtain a “ready to screen” bacterial collection from a marine rock pond. The continuous improvement of culture dependent methods and the efforts being made to cultivate marine bacteria will, in the future, result in sound biotechnological applications. The diversity of bacteria in marine habitats is tremendous, and unlocking their biological potential may lead to discoveries essential to reach the United Nations’ Sustainable Development Goals (Akinsemolu, 2018). Whole cell biocatalysts (de Carvalho, 2017) and enzymes (Ferrer et al., 2018), and strains able to produce secondary metabolites (de Carvalho and Fernandes, 2010), should be investigated for their biotechnological potential which may result in the production of e.g., pharmaceuticals, fine chemicals and agrochemicals.

## Data Availability

The datasets presented in this study can be found in online repositories. The names of the repository/repositories and accession number(s) can be found below: https://doi.org/10.5281/zenodo.6624673, doi: 10.5281/zenodo.6624673.
