# Supplementary material for: Cultivating marine bacteria under laboratory conditions: Overcoming the “unculturable” dogma
Source: Front Bioeng Biotechnol. 2022 Aug 17;10:964589. doi: 10.3389/fbioe.2022.964589 (PMC9428589; doi:10.3389/fbioe.2022.964589)
Supplement: Supplementary file 1 [file DataSheet1.DOCX]

Supplementary Material

#
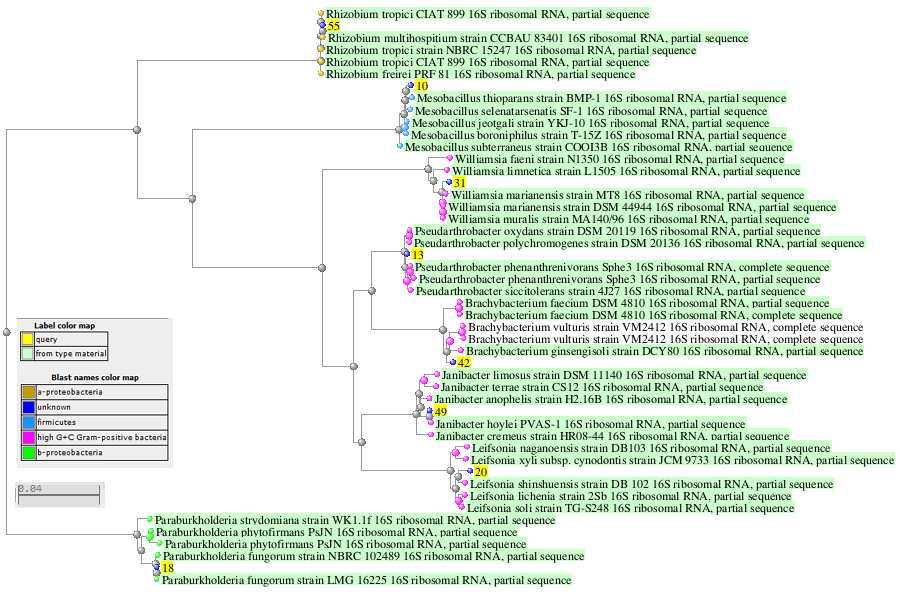


# Supplementary Figure 1 – Phylogenetic relationship between selected isolates and isolates deposited in GenBank. The Joining Neighbor Tree was done using MOLE-BLAST of NCBI.

# Supplementary Data

The dataset presented in this study can be found in an online repository at https://doi.org/10.5281/zenodo.6624673
